# Functionalized MoS_2_ Nanosheets as Multi-Gene Delivery Vehicles for *In Vivo* Pancreatic Cancer Therapy: Erratum

**DOI:** 10.7150/ntno.105822

**Published:** 2024-11-02

**Authors:** Feng Yin, Tommy Anderson, Nishtha Panwar, Kang Zhang, Swee Chuan Tjin, Beng Koon Ng, Ho Sup Yoon, Junle Qu, Ken-Tye Yong

**Affiliations:** 1 School of Chemical Biology and Biotechnology, Peking University Shenzhen Graduate School, Shenzhen, 518055, China; 2 School of Electrical and Electronic Engineering, Nanyang Technological University, Singapore 639798, Singapore; 3 Division of Structural Biology & Biochemistry, School of Biological Sciences, Nanyang Technological University, Singapore 639798, Singapore; 4 Key Laboratory of Optoelectronic Devices and Systems of Ministry of Education and Guangdong Province, College of Optoelectronic Engineering, Shenzhen University, Shenzhen, 518060, People's Republic of China

The authors would like to address an inadvertent error in the original version of the paper. Unfortunately, due to unforeseen circumstances, there was a misplacement of images in Figures 5A and 6A. Specifically, the image intended for the control of FA/MoS2 at 4 hours in Figure 5A was mistakenly repeated as the control image for FA/MoS2 (NIR). Similarly, the control image for FA/MoS2 (NIR) at 4 hours in Figure 6A was duplicated as the control for FA/MoS2/siHDAC1.

We sincerely apologize for this human error and any confusion it may have caused. The revised versions of Figures 5A and 6A are now provided below for clarity. Importantly, these corrections do not affect the original data or conclusions of the study.

Thank you for your understanding and patience as we work to ensure the accuracy of our research.

## Figures and Tables

**Figure 5A:**
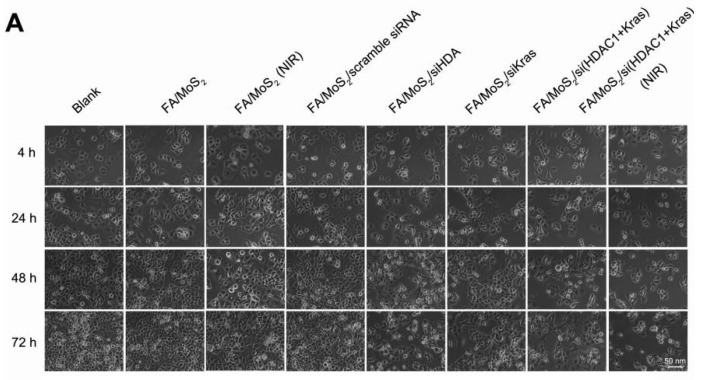
Cell viability tests of different MoS_2_-based formulations. The growth of Panc-1 cells is inhibited by FA/MoS_2_/siRNA nanocomplex with NIR light. Panc-1 cells are treated with PBS, FA/MoS_2_, FA/MoS_2_ with NIR light, FA/MoS_2_/scramble siRNA, FA/MoS_2_/siKRAS, FA/MoS_2_/siHDAC1, FA/MoS_2_/si(KRAS+HDAC1) and FA/MoS_2_/si(KRAS+HDAC1) with NIR light for 4 hours, then all the cells are washed with PBS and re-incubated in fresh cell medium for designated time. Phase contrast microscope images (A) and relative cell viabilities (B) of Panc-1 cells treated with different MoS_2_/siRNA nanocomplex formulations without or with NIR light for 4, 24, 48, and 72 hours. Data are presented as the means±SEM of triplicate experiment.*, P < 0.05, **, P < 0.01 vs PBS (as blank) and FA/MoS_2_.

**Figure 6A:**
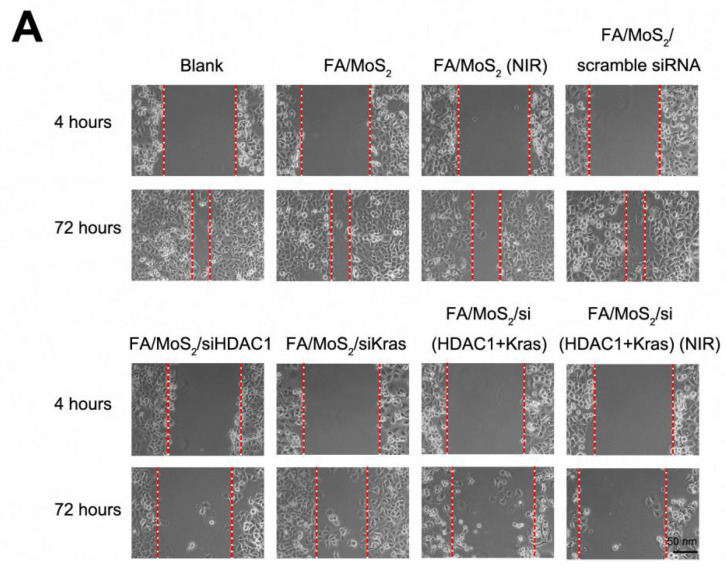
Wound healing migration assay in Panc-1 cell culture treated with different MoS_2_-based nanoparticle formulations. (A) Phase contrast microscope images of the wound healing process monitored Panc-1 cells treated with PBS, FA/MoS_2_, FA/MoS_2_ with NIR light, FA/MoS_2_/scramble siRNA, FA/MoS_2_/siKRAS, FA/MoS_2_/siHDAC1, FA/MoS_2_/si(KRAS+HDAC1) and FA/MoS_2_/si(KRAS+HDAC1) with NIR light at 4 and 72 hours. The gap is produced by scraping of the cell monolayer with a 200-μm micropipette tip. (B) Quantitative evaluation by measuring the width of the gap distance after treatment, values are normalized by the 4 hours wound gap width (as initial width). Data are presented as the means±SEM of triplicate experiments. **, P<0.01 vs PBS (as blank) and FA/MoS_2_.

